# The Association Between the Progressive Motility of Bovine Spermatozoa and the Developmental Morphokinetics of In Vitro-Derived Embryos

**DOI:** 10.3390/jdb14020024

**Published:** 2026-05-20

**Authors:** Shir Maizus, Dorit Kalo, Tanya Kogan, Ariel Michaelov, Zvi Roth

**Affiliations:** 1 Department of Animal Sciences, Robert H. Smith Faculty of Agriculture, Food and Environment, The Hebrew University, Rehovot 7610001, Israel; 2 Sion Artificial Insemination Center, Gedera 7057102, Israel

**Keywords:** bull fertility, embryo development, morphokinetics, progressive motility, spermatozoa morphology

## Abstract

The proportion of spermatozoa with progressive motility is widely used to evaluate the quality of a single ejaculate. However, the cellular and physiological mechanisms underlying this trait remain unclear. The present study examined the association between the progressive motility of bovine spermatozoa, their quality and their fertilization competence in vitro, and subsequently the association with the developmental morphokinetics of the formed embryos. Fresh ejaculates were classified and divided into groups with high (HPM), medium (MPM), or low (LPM) progressive motility. Then, spermatozoa were evaluated for their morphology, plasma membrane integrity, mitochondrial membrane potential, oxidative status, and acrosome integrity. The findings revealed that spermatozoa from HPM ejaculates enhanced motility in association with higher mitochondrial membrane potential relative to the LPM group, suggesting higher metabolic potential. No differences were recorded in fertilization competence among groups; however, the developmental kinetics of the formed embryos, determined by a time-lapse system, differed; embryos derived from HPM spermatozoa cleaved earlier to the two-, three-, and four-cell stages than embryos derived from MPM spermatozoa, suggesting that HPM-derived embryos are of good quality. Our findings suggest that progressive motility is not only a motility characteristic; it also reflects cellular quality of spermatozoa and the formed embryo.

## 1. Introduction

The reproductive performance of dairy herds has declined in recent decades [1,2,3]. While most of the studies have focused on cow health and fertility [4,5,6], less attention has been given to bull fertility [7,8]. Under intensive reproductive management, evaluating ejaculate quality is of high importance, since the semen of a single bull is usually used to fertilize hundreds to thousands of cows.

Conventionally, evaluation of ejaculate quality is based on spermatozoa concentration, motility, and morphology [9,10], mainly by utilizing computer-assisted spermatozoa analysis (CASA) [11]. Among these features, motility and particularly progressive motility have become key indicators of ejaculate quality. Progressive motility is defined as the ability of the spermatozoa to move forward linearly, an essential movement pattern through the female reproductive tract to reach and fertilize the ovulated oocyte. A positive relationship between the proportion of progressively motile spermatozoa and the fertility outcomes was previously reported by our group [12]. However, the physiological and cellular mechanisms underlying the acquisition and maintenance of progressive motility remain to be clarified.

The cellular structure of the spermatozoon, along with the functional integrity of its compartment, are required for successful fertilization; any impairment in these components may compromise spermatozoa function. Therefore, an accurate evaluation of ejaculate quality is particularly critical. A few techniques, such as fluorescence microscopy or flow cytometry, are used to evaluate the cellular features of spermatozoa [13]. The assessments include membrane integrity [13], mitochondrial membrane potential [13,14], acrosome integrity [15], DNA fragmentation [13,16,17], and membrane fluidity [17,18]. More recent technologies enable deeper analysis, such as proteomic, transcriptomic, and lipidomic analyses [12,19,20]. Moreover, studies in which cytokines and extracellular vesicles of the ejaculate were analyzed provide a broader view of the natural surrounding environment of spermatozoa [21,22]. Nevertheless, assessment of ejaculate quality not always provides a 100% prediction of spermatozoa fertility success.

Fertility competence, which includes spermatozoa binding to the zona pellucida and zygote formation, is considered a diagnostic test that predicts the fertilizing potential of spermatozoa [23]. Using an in vitro fertilization (IVF) approach provides a powerful platform to study fertilization potential of the spermatozoa and developmental competence of the formed embryo. Moreover, previous studies imply that spermatozoa quality can affect early development of embryos [24], in particular, the quality of the developed blastocyst [19]. With respect to the latter, time-lapse imaging systems that enable continuous assessment of embryo morphokinetics, morphology and the accurate timing of key developmental events, have been suggested to evaluate embryo quality and developmental potential for a broader view [25,26]. Nonetheless, the direct impact of spermatozoa progressive motility on embryo developmental morphokinetics is less known.

The present study aimed to overcome this gap; it examined three different progressive motility levels, i.e., ejaculates with high (HPM), medium (MPM), and low (LPM) progressive motility, and their association with (1) spermatozoa morphology, motility dynamics and structural integrity features; (2) fertilization and developmental competence in vitro; and (3) developmental morphokinetics of in vitro-derived embryos. Collectively, this approach might provide a comprehensive assessment of progressive motility as a biological feature and its relevance to reproductive outcomes.

## 2. Materials and Methods

### 2.1. Experimental Design

All experiments were performed during the cold season to avoid any thermal stress on spermatozoa and/or oocytes. A total of 119 fresh ejaculates were collected from *n* = 58 Holstein–Friesian working bulls and classified into three groups, high (HPM; *n* = 55), medium (MPM; *n* = 38), and low (LPM; *n* = 26), according to the proportion of progressive motility spermatozoa within the ejaculate. Ejaculate grouping was based on CASA thresholds, as was previously reported by our group [12]. For all experimental groups, the ejaculate rather the bull itself was considered the experimental unit. Each ejaculate was processed and evaluated independently under identical conditions.

**Figure 1:**
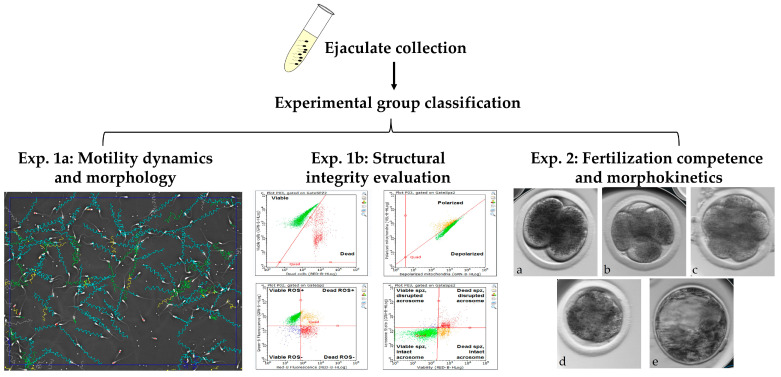
Schematic presentation of the experimental workflow. Ejaculates were collected from bulls and divided into 3 different experimental groups based on their progressive motility: high progressive motility (HPM), medium progressive motility (MPM), and low progressive motility (LPM). Each sample underwent a comprehensive evaluation encompassing three analytical tests: Experiment 1a (Exp. 1a) included a motility dynamic and morphology evaluation using IVOS computer-assisted sperm analysis (CASA). In Experiment 1b (Exp. 1b), flow cytometry was used to quantify key structural features (viability, reactive oxygen species (ROS) levels, mitochondrial membrane potential, and acrosome integrity). Scatter plots depict the distribution of spermatozoa across relevant physiological states (e.g., viable vs. dead, polarized vs. depolarized, ROS-positive vs. ROS-negative, and acrosome-reacted vs. intact). Experiment 2 (Exp. 2) was an assessment of fertilization competence and embryo developmental morphokinetics via in vitro fertilization. Images a–e illustrate the embryonic stages.

The experimental design consisted of two experiments (Figure 1): Experiment 1 aimed to examine the associations between spermatozoa progressive motility and morphological and motility dynamics (Experiment 1a) and between progressive motility structural integrity features (Experiment 1b). In Experiment 1a we evaluated the motility and morphology of spermatozoa in each experimental group (HPM, MPM, and LPM) by using IVOS computer-assisted sperm analysis (CASA). In Experiment 1b we evaluated structural integrity, i.e., viability, oxidation status, mitochondrial membrane potential, and acrosome integrity, using an EasyCyte flow cytometer.

Experiment 2 aimed to determine an association between progressive motility and fertilization competence (Experiment 2a) and the embryo developmental morphokinetics (Experiment 2b). For Experiment 2a we used the in vitro embryo production model; fertilization was performed with freshly collected ejaculates (HPM, MPM, and LPM) and cumulus–oocyte complexes (COCs; *n* = 814); in vitro culture was performed in a conventional incubator. Experiment 2b included a similar in vitro embryo production methodology with minor modifications; the putative zygotes were cultured individually in an incubator equipped with a time-lapse system; this approach enabled us to record the developmental morphokinetics of the embryos in real time. This experiment included a total of *n* = 389 COCs. It should be noted that the aspirated COCs were randomly divided into the experimental groups, in order to minimize potential bias and to ensure that any variability in oocyte quality was evenly distributed among groups.

### 2.2. Ejaculate Collection

Ejaculates were collected from Holstein–Friesian working bulls at SION Ltd. (Hafetz Haim, Israel). Bulls were stimulated to mount a teaser-bull, and ejaculates were collected using a sterile artificial vagina pre-warmed to 38 °C as described by Orgal et al., 2012 [27]. The ejaculates were maintained at 37 °C until analysis. All the procedures were conducted in accordance with the ethical guidelines of the Hebrew University of Jerusalem and were approved by the institutional ethics committee for the use of biological models (AG-22-16883-1).

### 2.3. Motility Dynamic and Morphological Evaluation

**Figure 2:**
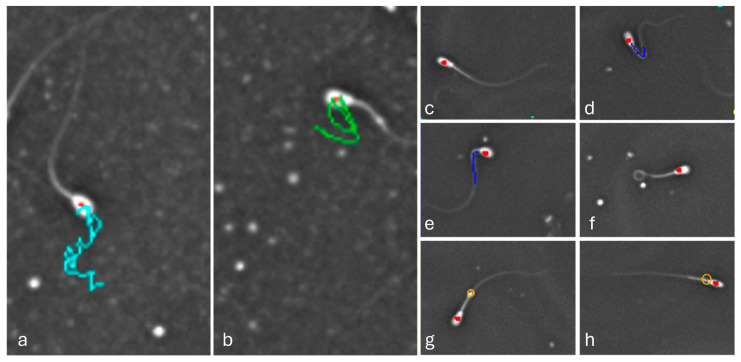
Representative snapshots taken from IVOS II. (**a**) Spermatozoa (highlighted by a red dot) representing progressive motility, with the motility path indicated by a light blue line; (**b**) spermatozoa representing circulated motility, highlighted by a green line; (**c**) spermatozoa representing a normal morphology; (**d**) spermatozoa with a bent tail, highlighted by blue line; (**e**) spermatozoa with a bent structure from the midpiece, highlighted by blue line; (**f**) spermatozoa with a coiled tail; (**g**) spermatozoa with a distal cytoplasmatic droplet; and (**h**) spermatozoa with a proximal droplet, highlighted by orange circles.

The collected ejaculates were immediately analyzed using the IVOS II CASA system (Hamilton Thorne Biosciences, Beverly, MA, USA), calibrated for bovine spermatozoa [12]. For that purpose, the ejaculates were gently mixed and loaded into pre-warmed analysis chambers (37 °C). Ejaculate analysis was performed under standardized illumination and optical settings using a Zeiss ×10 negative phase contrast objective. Illumination was provided by a red LED light source with constant photometer settings (60–70) and was kept constant for all measurements. For each sample, at least eight non-overlapping fields were recorded to ensure a representative assessment. The measured parameters included the total number and the proportion of progressive motility (PM) spermatozoa and velocity average path (VAP), as well as structural abnormalities (bent/coiled tails, distal/proximal droplets, and distal midpiece reflex; Figure 2).

Progressive motility was defined as the proportion of spermatozoa exhibiting forward-linear movement. The established progressive motility thresholds were used to classify the ejaculates into three groups using high (HPM; ≥74%), medium (MPM; 60–73.9%), and low (LPM; <60%) progressive motility.

### 2.4. Spermatozoa Cellular Evaluation

From each ejaculate, a sample of 50 µL was washed twice in NKM (110 mM NaCl, 5 mM KCl, and 20 mM MOPS [3-N-morphilino propanesulfonic acid, pH 7.4]) buffer and underwent the swim-up procedure, i.e., incubated at 37 °C for 15 min at a 30° angle [28]. Then, spermatozoa vitality and motility were confirmed under a microscope, followed by cellular trait evaluation using an EasyCyte flow cytometer (IMV Technologies, L’Aigle, France) with bovine-dedicated commercial kits, as was previously reported [12,13]. Analyses included assessment of viability, reactive oxygen species (ROS) level, mitochondrial membrane potential, and acrosome integrity.

Plasma membrane integrity was evaluated using EASYKIT 1 (IMV Technologies). The kit contains two fluorochromes and enables to distinguish between viable and dead spermatozoa. The results were presented as the percentage of viable spermatozoa.

Acrosome membrane integrity was evaluated with EASYKIT 3 (IMV Technologies). The kit enables to distinguish between viable and dead spermatozoa with either intact or damaged acrosome. The results were presented as the percentage of viable spermatozoa with an intact acrosomal membrane out of total cells.

The oxidation status was evaluated with EASYKIT 3 (IMV Technologies). The kit enables to distinguish between viable and dead spermatozoa that either express or do not express reactive oxygen species (ROS). The results were presented as the percentage of viable spermatozoa with ROS out of total cells.

The mitochondrial membrane potential of the spermatozoa was evaluated using EASYKIT 2. The kit enables to distinguish between spermatozoa with either polarized (i.e., high mitochondrial membrane potential) or depolarized (i.e., low mitochondrial membrane potential) mitochondrial membrane. The results were expressed as the ratio between the percentages of spermatozoa expressing polarized relative to depolarized mitochondrial membranes.

For each assay, two technical replicates of 5000 spermatozoa per sample were acquired. All kits were evaluated simultaneously without any further sample storage.

### 2.5. In Vitro Embryo Production

The in vitro embryo production (IVF) procedure, a well-established methodology in our lab, was performed as previously described [19,26,29]. Chemicals were obtained from Merck-Sigma unless otherwise specified. Oocyte maturation medium (OMM), HEPES-buffered tyrode’s Albumin lactate pyruvate (HEPES-TALP), spermatozoa preparation TALP (SP-TALP), in vitro fertilization TALP media (IVF-TALP), and potassium-modified simplex optimization medium (KSOM), were prepared according to standard protocols.

Ovaries from lactating Holstein cows were collected at the slaughterhouse and transported to the lab within 2 h in warm saline (38.5 °C) supplemented with penicillin–streptomycin. In the lab, follicles (3–8 mm) were aspirated; then cumulus–oocyte complexes (COCs) with homogenous cytoplasm and multiple compact cumulus layers were selected, washed, and matured in vitro (30–40 COCs per well) for 22 h at 38.5 °C in 5% CO_2_. In vitro fertilization was performed with fresh ejaculates; ejaculates were washed in SP-TALP (600× *g*) and IVF-TALP (200× *g*) and then subjected to the swim-up procedure. Volumes of 30 µL of motile spermatozoa suspension were co-cultured with the COCs to enable fertilization, with the addition of 25 µL of PHE solution (penicillamine, hypotaurine, and epinephrine) for 18 h at 38.5 °C in 5% CO_2_. Then, the putative zygotes were denuded from cumulus cells by brief vortexing, washed, and cultured in KSOM (10 zygotes per 25 µL dropped into mineral oil) for 8 days in a conventional incubator (Exp. 2a; 38.5 °C in 5% CO_2_ and 5% O_2_). Spermatozoa fertilization competence was assessed as the proportion of embryos that cleaved to the 2–4-cell stages 42–44 h post-fertilization. In addition, the blastocyst formation rate on days 7–8 post-fertilization, was calculated relative to the total COCs or cleaved embryos.

#### Developmental Morphokinetics

A subset of putative zygotes was cultured in a Miri^®^ time-lapse incubator (Esco Medical Technologies Pte, Ltd., Kringelled, Denmark) for continuous monitoring over 190 h, as was reported previously (Exp. 2b; [26]). Each putative zygote was cultured individually in 25 µL KSOM drops in a CultureCoin dish (Esco Medical) in mineral oil at 38.5 °C in 5% CO_2_ and 5% O_2_. Images were acquired every 5 min through seven focal planes (20× Zeiss objective, NA 0.35, 635 nm). Embryo developmental morphokinetics were assessed by recording the timing of key developmental events, including cleavage to the 2-, 3-, and 4-cell stages, morula formation, and blastocyst formation and expansion, throughout the 190 h post-fertilization period. Embryos were classified as normal or abnormal according to the pattern of the first cleavage. Normally cleaved embryos expressed symmetric blastomeres; abnormally cleaved embryos expressed direct cleavage (i.e., from one blastomere to three or more blastomeres), unequal cleavage (i.e., into two unevenly sized blastomeres), and reverse cleavage (i.e., reduced number of blastomeres, from two to one).

### 2.6. Statistical Analysis

All data were analyzed using JMP Pro 18 software (SAS Institute, Inc., Cary, NC, USA). A comparison of motility dynamics, morphology, structural integrity, cleavage and blastocyst formation rates among the experimental groups was subjected to one-way ANOVA, followed by the Tukey–Kramer test. Before that, the proportion of cleavage to 2- to 4-cell-stage embryos and the blastocyst formation rate were arcsine-transformed. Non-parametric data, including embryo developmental timing (morphokinetics), were analyzed using the Kruskal–Wallis test, followed by pairwise Wilcoxon comparisons. Categorical data were analyzed using the chi-square test, followed by the Pearson’s or Fisher’s exact test, as appropriate. Data are presented as the means ± SEMs unless otherwise stated. For all analyses, *p* < 0.05 was considered statistically significant, whereas *p*-values between 0.05 and 0.10 were reported as trends. All experiments included at least three independent biological replicates.

## 3. Results

### 3.1. Experiment 1: Motility Dynamics, Morphological and Structural Characteristics

#### 3.1.1. Experiment 1a: Motility Dynamics and Morphological Characteristics of Spermatozoa

Ejaculates differed among the progressive motility groups in several motility and morphology parameters (Table 1). Spermatozoa from the LPM and MPM samples expressed a higher proportion of spermatozoa with bent and coiled tails and a higher proportion of spermatozoa with distal cytoplasmic droplets relative to the spermatozoa of the HPM group. In addition, the spermatozoa from the HPM group displayed a higher proportion of morphologically normal spermatozoa relative to the MPM and LPM groups (Table 1: *p* ≤ 0.01). Motility-related traits exhibited a consistent gradient across groups, with all pairwise comparisons indicating highly significant progressive motility (HPM > MPM > LPM) (Table 1; *p* < 0.0001). As expected, the observed progressive motility values corresponded to the predefined classification threshold (Table 1).

#### 3.1.2. Experiment 1b: Structural Integrity Characteristics of Spermatozoa

**Figure 3:**
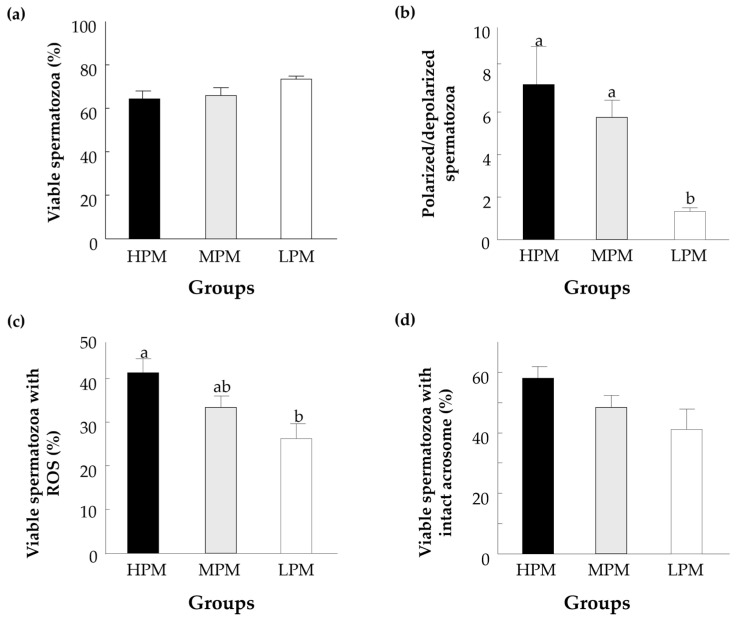
Structural analysis of spermatozoa with high (HPM), medium (MPM), and low (LPM) progressive motility. Presented are (**a**) the proportion of spermatozoa classified as viable, (**b**) the ratio of mitochondrial membrane potential (polarized/depolarized), (**c**) the proportion of live spermatozoa that expressed reactive oxygen species (ROS), and (**d**) the proportion of live spermatozoa with an intact acrosome membrane. Data are presented as the means ± SEMs calculated for at least 3 replicates with 5000 spermatozoa per replicate. The different letters above indicate significant differences between the experimental groups (*p* < 0.05).

Overall, spermatozoa from the HPM group exhibited superior morphology, motility performance, and mitochondrial function parameters, underscoring progressive motility as a robust and integrated indicator of spermatozoa quality (Figure 3). Cellular analyses revealed that the proportion of viable spermatozoa did not differ among groups (Figure 3a). On the other hand, the proportion of spermatozoa that exhibited higher mitochondrial polarization was higher in the HPM relative to the LPM group (Figure 3b; *p* = 0.04). Moreover, the HPM group exhibited a higher proportion of viable spermatozoa with ROS positivity following the induction of oxidative stress (Figure 3c; *p* = 0.01). No differences were observed in the proportion of viable spermatozoa with an intact acrosome membrane among groups (Figure 3d).

### 3.2. Experiment 2: Fertilization Outcomes and Embryo Morphokinetics

#### 3.2.1. Experiment 2a: Fertilization Outcomes

Putative zygotes were cultured in a conventional incubator; the findings revealed that cleavage rates to the two- and four-cell stages (~40 h post-fertilization) did not differ among the progressive motility groups (Table 2). In addition, the blastocyst formation rates did not differ among groups neither when the proportion of the developed blastocysts was calculated out of total oocytes nor when it was calculated out of cleaved embryos (Table 2).

#### 3.2.2. Experiment 2b: Embryo Developmental Morphokinetics

Putative zygotes were cultured in an incubator equipped with a time-lapse system. Fertilization competence, expressed by the proportion of cleaved embryos (~40 hpf), did not differ among groups (Table 3). A tendency of increase was found in the cleavage rate between the MPM and LPM groups (*p* = 0.09). No differences were found between the LPM and HPM groups. In addition, the blastocyst formation rates did not differ among groups when the proportion of developed blastocysts was calculated out of total oocytes or when it was calculated out of cleaved embryos.

**Figure 4:**
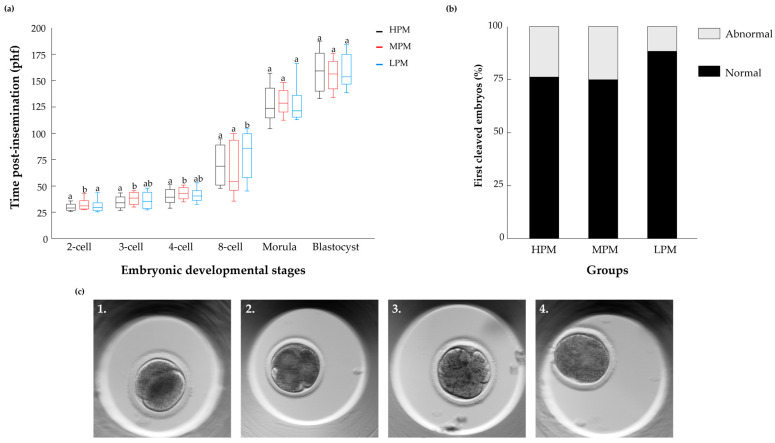
Morphokinetics of embryos that developed from the HPM, MPM and LPM groups. (**a**) The box-and-whisker plot represents the embryonic developmental kinetics from all cleaved embryos. The *x*-axis displays the embryonic developmental stage (from 2-cell embryo to blastocyst), and the *y*-axis indicates hours post-insemination (hpf) at which embryos reached each respective stage. Boxes represent the interquartile range; the horizontal line inside each box indicates the median, and the whiskers indicate the full data range. Statistical analysis was performed using the Kruskal–Wallis test, followed by the Wilcoxon post hoc test. Different letters above the boxes indicate statistically significant differences between groups (*p* < 0.05). (**b**) Embryo distribution according to their cleavage pattern into the normal and abnormal categories. Pearson’s chi-square test was performed to evaluate differences in embryo development rate among groups. (**c**) Representative images of first-cleavage patterns: (**1**) normal cleavage (**2**) direct cleavage into 3 blastomeres, (**3**) unequal cleavage and (**4**) reversed cleavage. The different letters above each box indicate significant differences between the experimental groups within each embryonic developmental stage (*p* < 0.05).

Time-lapse analysis revealed differences in embryonic developmental kinetics among the progressive motility groups. Embryos that developed following fertilization with HPM spermatozoa cleaved earlier through the first divisions to the two-, three-, and four-cell stages relative to embryos that derived from MPM spermatozoa (*p* ≤ 0.05; Figure 4a). In addition, embryos that developed following fertilization with HPM or MPM spermatozoa cleaved earlier to the eight-cell stage relative to embryos that derived from LPM spermatozoa (*p* = 0.05).

Embryos were classified according to the pattern of the first cleavage as normal or abnormal (i.e., direct, unequal, or reverse divisions; Figure 4b,c). The findings revealed that the pattern of the first embryonic division (Figure 4b; *p* = 0.7) did not differ among the groups. In particular, the proportions of normally cleaved embryos were 76.3, 75.0, and 82.4% for the HPM, MPM, and LPM groups, respectively.

With respect to the abnormal cleavage categories, no significant differences were observed among groups. The proportions of embryos undergoing direct cleavage were 47.4, 30.8 and 16.7% for the HPM, MPM, and LPM groups, respectively. The proportions of reversed cleaved embryos were 18.5, 19.2 and 33.3% for the HPM, MPM, and LPM groups, respectively. The proportions of unequally cleaved embryos 34.2, 50.0 and 50.0% for the HPM, MPM, and LPM groups, respectively. It should be noted that only a very small number of embryos developed in the LPM group; therefore, these results should be interpreted with caution.

## 4. Discussion

Progressive motility reflects the ability of spermatozoa to move forward; it is one of the commonly used parameters for evaluating ejaculate quality. Here we report that the proportion of progressively motile spermatozoa within the ejaculate (i.e., high, medium, or low) is associated not only with morphological and kinetic characteristics but also with the cellular traits of the spermatozoa; this might explain, at least in part, the mechanisms that underlie the progressive pattern. In addition, the findings provide new evidence that associates progressive motility with the developmental kinetics of the formed embryo. Although the fertilization and developmental rates did not differ among groups with high, medium, and low progressive motility, embryos that were derived from HPM spermatozoa cleaved earlier through the first mitotic divisions. Early-cleaved embryos have been shown to have high developmental competence [26].

Previous studies reported that the ability of spermatozoa to move progressively largely depends on their morphology [7,30]. In support of this, our findings indicate that highly progressively motile ejaculates expressed a higher proportion of spermatozoa with normal morphology, suggesting a higher degree of cellular and structural maturation through spermatogenesis. On the other hand, the relatively high incidence of coiled or bent tails and retained cytoplasmic droplets in the MPM and LPM ejaculates, reported here, might result from incomplete maturation. These alterations are known to be associated with impaired movement and reduced fertilization potential [31,32,33]. Tail abnormalities such as coiled or bent flagella are known to disrupt the flagella symmetry, thereby altering progressive movement. Similarly, retained cytoplasmic droplets are widely considered a marker for incomplete epididymal maturation and have been associated with altered membrane composition, increased oxidative stress, and compromised motility [34,35]. In addition, a previous study reported that differential changes in the morphological parameters led to changes in motility and velocity [36]. Spermatozoa velocity reflects the efficiency of flagellar beating, which depends on an intact axonemal structure and an adequate ATP supply. Given that in the present study high progressive motility was associated with high velocity, it is reasonable to assume that both patterns result from mitochondrial function and high ATP production. Taken together, the findings support the notion that the proportion of progressively motile spermatozoa within ejaculate is associated with morphologic and kinematic characteristics; however, they are also tightly linked to intrinsic cellular functions. Consequently, progressive motility should be considered not only to be a motion pattern but also to reflect intracellular processes that maintain spermatozoa function.

Mitochondria play a central role in spermatozoa activities, since they provide the energy for their movement [37]. Here we found that spermatozoa from HPM ejaculates exhibited a higher mitochondrial membrane potential, a key parameter for ATP production through the phosphorylation cascade, thus reflecting the cell’s energetic capacity [38]. We suggest that the ability to generate energy not only supports spermatozoa motility [39,40] but also contributes, at least in part, to the acquisition and maintenance of a progressively motile pattern. In support of this, previous work with cryopreserved ejaculate showed that HPM spermatozoa have higher mitochondrial membrane potential (ΔΨm) compared with LPM spermatozoa [41]. Similarly, a study in humans reported a close association between increased progressive motility and elevated mitochondrial membrane potential [42]. Another study reported that mitochondrial respiratory efficiency is positively correlated with spermatozoa motility [43]. Taken together, the association between mitochondrial function and progressive motility reported here further supports the notion that mitochondrial activity regulates spermatozoa motility.

The higher mitochondrial polarity observed in HPM spermatozoa was accompanied by a significantly lower incidence of DMR i.e., the distal midpiece reflex. On the other hand, the LPM and MPM ejaculates displayed a higher prevalence of DMR and reduced mitochondrial membrane potential. The DMR is a common abnormality in bull spermatozoa where the tail bends sharply at the junction with the spermatozoon midpiece region, where the mitochondria are located. Structural abnormalities in this region are likely to compromise mitochondrial organization and respiratory efficiency. Accordingly, it is suggested that the high DMR rates observed in the LPM group reflect a mechanistic link between midpiece structural alterations, which in turn, might impair mitochondrial function and progressive motility. This interpretation is supported by studies in humans that associated spermatozoa midpiece defects to disruption in mitochondrial activity and compromised spermatozoa motile performance [43,44,45]. Collectively, these observations suggest that progressive motility integrates both mitochondrial energetic competence and the structural integrity of the midpiece region; thus, disruption of this functional axis may underlie the reduced progressive motility observed in LPM and MPM ejaculates.

In accordance with the higher mitochondrial membrane potential, a higher proportion of viable spermatozoa expressing ROS was recorded in the HPM group. Since the mitochondrial membrane potential correlates with mitochondrial activity [46], it is possible that HPM spermatozoa have more active mitochondria, which is expressed by greater ROS production. ROS are known to play a dual role in regulating spermatozoa activities. At physiological levels, ROS are essential mediators of fertilization-related processes, including capacitation, hyperactivation, and tyrosine phosphorylation cascades, which are known to regulate flagellar activity and spermatozoa motility [47]. ROS expression at physiological levels reflects elevated metabolic processes and functional activities [48,49]. On the other hand, excessive or uncontrolled ROS generation can lead to lipid peroxidation or protein oxidation, which in turn causes mitochondrial dysfunction and DNA damage, which in turn can ultimately impair spermatozoa viability and fertilizing capacity [47,50]. In the current study, spermatozoa viability did not differ among groups; therefore, it is reasonable to assume that the higher proportion of spermatozoa expressing ROS observed in the HPM group expresses a normal or balanced oxidative status rather than oxidative stress.

Successful fertilization and embryonic development depend on the quality and competence of both the oocyte and the spermatozoon, as each contributes essential cellular and molecular components required for development. While there is a broad consensus that oocyte quality is a primary determinant of embryonic developmental, spermatozoa have been also suggested to play a significant role in fertilization outcomes and early embryo development [51]. For the current study, we assumed that the superior motility and morphology characteristics observed in HPM spermatozoa would also be reflected in higher fertilization competence. However, neither cleavage to the two- or four-cell stages nor the development to the blastocyst stage differed among the progressive motility groups. This was true for the two-culture methodology used in the current study (i.e., culturing in a conventional incubator or in incubators equipped with a time-lapse system). One possible explanation is similar fertilization competence among the progressive motility groups. In support of this assumption are the findings that spermatozoa viability and acrosome membrane integrity did not differ among the progressive motility groups. Acrosome membrane integrity is positively associated with spermatozoa fertilization competence [15,52,53]. Moreover, the acrosome status is important for capacitation assessment [15]. In contrast to our findings, an in vitro fertilization study with cryopreserved semen reported that although acrosome integrity did not differ between groups, the proportions of embryos that cleaved to two- and four-cell stages and developed to blastocysts were higher in the HPM group than in the LPM group [41]. The discrepancies between studies may be related to the source of the spermatozoa used for fertilization, cryopreserved vs. fresh semen as used in the current study. Another option might be a different progressive motility cutoff; Chapuis et al. [54] reported a higher fertilization rate when progressive motility was >32%. In addition, one might suggest that within the spermatozoa subpopulation (e.g., the LPM group), the spermatozoa that ultimately participate in fertilization may still possess sufficient functional competence. Another possible explanation is the experimental model (i.e., in vitro vs. in vivo). In the current study we used an in vitro fertilization model; therefore, it is possible that HPM spermatozoa did not have any advantage in their progressive movement but might have benefits in vivo. In support of this assumption, artificial insemination with frozen–thawed straws containing 6 million progressively motile spermatozoa/straw resulted in higher conception rates relative to insemination with straws containing 1.5 million progressively motile spermatozoa [12]. In support of this, a previous in vivo study in stallions reported a positive association between progressive motility and pregnancy rate [55]. Nevertheless, it should be noted that an in vitro embryo production system enabled the association between intrinsic parameters that correlate with progressively motile spermatozoa with early embryonic development, during the first days following fertilization, which cannot be obtained in field experiments. Here, we provide the first piece of evidence that spermatozoa progressive motility is associated with the developmental kinetics of the formed embryo.

Interestingly, although the embryo developmental rate did not differ among groups, the morphokinetic analysis revealed that embryos derived from HPM spermatozoa cleaved earlier during the first three mitotic divisions compared with embryos derived from the MPM group. In a previous study we documented that the timing of the first three divisions of the cleaved embryo is associated with its developmental competence [26]. Moreover, Sugimura et al. [56] reported that early-cleaved embryos have higher implantation competence. Based on human studies, there is a strong association between cleavage timing and pregnancy or conception success; embryos that cleaved earlier in the first divisions expressed a higher conception rate [57,58]. However, this point remains unclear in bovines and deserves further examination.

Our findings support the broader concept that paternal factors carried by the spermatozoa may influence embryonic developmental dynamics [59]. In support of this, a previous human study found that spermatozoa motility is highly correlated with the timing of the pronuclei fading and the two-cell stage formation, while spermatozoa morphology is positively associated with the time of pronuclei fading and the formation time of four-cell stage embryos [60]. An additional human IVF study reported positive correlations between non-progressive spermatozoa motility and early embryo morphokinetics, including the timing of the two-, three- and four-cell cleavages [61]. Nonetheless, in the current study, despite marked differences in progressive motility among the experimental groups, embryos that derived from LPM spermatozoa did not statistically differ in their developmental kinetics relative to embryos derived from with HPM spermatozoa. While not examined here, these findings might reflect the role of oocyte competence as a key factor influencing embryo development.

## 5. Conclusions

The current study provides new insights into spermatozoa progressive motility as an acceptable marker for bull fertility. The findings suggest that differential progressive motility (i.e., high, medium, and low) between ejaculates also involves differences in the morphological and cellular traits. Our findings suggest that progressive motility, as a paternal factor, is associated with the developmental kinetics of the formed embryo. Nonetheless, further studies are required to elucidate the biological role of progressive motility and to investigate its potential to improve not only blastocyst formation but also implantation and successful pregnancy outcomes. Further examinations may include an embryo transfer trail, using in vitro-formed blastocysts derived from fertilization with HPM spermatozoa. Such examinations might confirm whether the spermatozoa progressive motility trait has additional effects on fertility outcomes. Alternatively, an in vivo fertility study using fresh HPM ejaculates should be conducted. In a previous fertility trial in lactating cows, we found that increasing the number of HPM spermatozoa within the insemination straw resulted in higher conception rates [12]. Nevertheless, in that study, the proportion of progressive motile spermatozoa was artificially made. Furthermore, during straw preparation, ejaculates were diluted and underwent cryopreservation. It is suggested, therefore, to perform a large-scale fertility trial to compare conception rate following artificial insemination with fresh HPM vs. LPM ejaculates. Taken together, the suggested studies may clarify whether differences in progressive motility are translated to fertilization success.

## Figures and Tables

**Table 1:** Comparing the motility and morphology parameters of spermatozoa among progressive motility groups.

Parameter	HPM	MPM	LPM
Bent tail (%)	0.42 ± 0.05 ^a^	0.86 ± 0.09 ^b^	1.23 ± 0.14 ^c^
Coiled tail (%)	0.18 ± 0.02 ^a^	0.39 ± 0.06 ^b^	0.36 ± 0.05 ^b^
Distal droplet (%)	1.87 ± 0.18 ^a^	1.98 ± 0.24 ^a^	3.14 ± 0.61 ^b^
DMR (%)	1.46 ± 0.10 ^a^	1.99 ± 0.14 ^b^	2.39 ± 0.19 ^b^
Normal morphology (%)	95.67 ± 0.26 ^a^	93.96 ± 0.45 ^b^	91.63 ±0.71 ^c^
Motile mean VAP (µm/s)	159.87 ± 2.01 ^a^	151.97 ± 2.66 ^b^	144.14 ± 3.70 ^b^
Motile (%)	93.09 ± 0.47 ^a^	87.67 ± 0.63 ^b^	82.19 ± 2.40 ^c^
Progressive mean VAP	165.69 ± 2.12 ^a^	161.97 ± 2.46 ^ab^	155.63 ± 3.13 ^b^
Progressive (%)	77.93 ± 0.7 ^a^	67.30 ± 0.82 ^b^	54.40 ± 1.05 ^c^

Data are presented as the means ± standard errors of the means (SEMs). HPM = high progressive motility; MPM = medium progressive motility; LPM = low progressive motility; DMR = distal midpiece reflex; VAP = motile mean velocity average path. ^abc^ Different superscript letters indicate significant differences for each parameter within the row, i.e., between experimental groups (*p* < 0.05).

**Table 2:** Comparison of the cleavage and blastocyst formation rates among HPM, MPM, and LPM groups (conventional incubator).

Parameters	HPM	MPM	LPM	*p*-Value
2- to 4-cell-stage embryos (%)	79.7 ± 3.9	72.5 ± 3.2	77.2 ± 8.0	0.5
Blastocysts out of total oocytes (%)	18.4 ± 4.5	15.9 ± 4.2	22.3 ± 4.2	0.3
Blastocysts out of total cleaved embryos (%)	22.0 ± 4.6	22.1 ± 5.8	26.0 ± 10.6	0.3

Analysis included *n* = 188, 226, and 136 cleaved embryos and *n* = 47, 52, and 53 blastocysts for HPM, MPM and LPM groups, respectively. Values are presented as the means ± standard errors of the means; HPM = high progressive motility; MPM = medium progressive motility; LPM = low progressive motility. Data were analyzed using one-way ANOVA followed by Tukey’s post hoc test; *p*-values indicate overall group differences.

**Table 3:** Comparison of cleavage and blastocyst formation rates among HPM, MPM, and LPM groups (incubator equipped with time-lapse system).

Parameters	HPM	MPM	LPM	*p*-Value
2- to 4-cell-stage embryos (%)	85.7 ± 1.1	90.0 ± 3.5	79.4 ± 2.4	0.09
Blastocysts out of the total oocytes (%)	20.0 ± 4.2	25.2 ± 3.3	21.8 ± 12.7	0.8
Blastocysts out of the total cleaved embryos (%)	23.5 ± 4.9	27.9 ± 2.9	28.1 ± 16.9	0.8

Analysis included *n* = 180, 117, and 38 cleaved embryos and *n* = 43, 33, and 11 blastocysts for HPM, MPM and LPM groups, respectively. Values are presented as the means ± standard errors of the means; HPM = high progressive motility; MPM = medium progressive motility; LPM = low progressive motility. Data were analyzed using one-way ANOVA followed by Tukey’s post hoc test; *p*-values indicate overall group differences.

## Data Availability

The data presented in this study are available upon request from the corresponding author.
